# Effects of acupuncture to treat fibromyalgia: A preliminary randomised controlled trial

**DOI:** 10.1186/1749-8546-5-11

**Published:** 2010-03-23

**Authors:** Kazunori Itoh, Hiroshi Kitakoji

**Affiliations:** 1 Department of Clinical Acupuncture and Moxibustion, Meiji University of Integrative Medicine, Hiyoshi-cho, Nantan, Kyoto 629-0392, Japan

## Abstract

**Background:**

Acupuncture is often used to treat fibromyalgia (FM), but it remains unclear whether acupuncture is effective. This study aims to evaluate the effects of acupuncture on pain and quality of life (QoL) in FM patients.

**Methods:**

Sixteen patients (13 women and 3 men aged 25-63 years) suffering from FM were randomised into two groups: group A (*n *= 8) received five acupuncture treatments after the fifth week and group B received ten acupuncture treatments. Outcome measures used in this study were pain intensity (visual analogue scale, VAS) and the fibromyalgia impact questionnaire (FIQ).

**Results:**

After the fifth week, pain intensity (*U *= 25.0; *P *= 0.022) in group B decreased and QoL (*U *= 24.5; *P *= 0.026) improved compared to group A.

**Conclusion:**

The present study suggests that acupuncture treatment is effective to relieve pain for FM patients in terms of QoL and FIQ.

## Background

Fibromyalgia (FM) is a condition of unknown cause; it is characterized by widespread musculoskeltal pain with symptoms including stiffness, fatigue, sleep disturbance and functional impairment [1,2]. According to the American College of Rheumatology criteria, an FM patient must have chronic widespread pain and at lest 11 out of 18 tender points on examination [1]. Affecting 2-4% of the populations in industrialized countries, FM is the second most common rheumatologic disorder in the world [3,4]. A wide range of treatment methods are currently in use such as medications, physical methods and manual treatments [5,6]. Complementary and alternative medicine (CAM) is commonly used to treat FM patients [7]. Ninety-one per cent (91%) FM patients used CAM [8] and significantly more FM patients used CAM compared to patients of other rheumatologic diseases [9]. One of the most commonly used forms of CAM is acupuncture (annual utilization percentages, from 4.8-6.7% of lifetime experiences to 19.4-26.7% in Japan) [10]. Although acupuncture has been used for pain relief for a long time in China and around the world, studies on the efficacy of acupuncture on FM provided mixed results [1,11]. Most studies which indicated beneficial effect of acupuncture treatment for FM were uncontrolled case series [1,11,12]; only a few supported that acupuncture was effective [1,13-16]. A recent systematic review found no evidence to show beneficial effects of acupuncture to treat FM compared with placebo [1]. Although efficacy of acupuncture have been assessed with various controls such as no-treatment controls [17], non-penetration needling [18], minimal acupuncture [19,20] and mock transcutaneous electrical nerve stimulation (TENS) [21,22], most studies on the efficacy of acupuncture for FM were uncontrolled case series that utilized electroacupuncture treatment [1,11], which make the setting of controls difficult because it involves perceptible current [23]. One of the alternative ideas which enable evaluation of the efficacy of acupuncture acceptable may be utilization of standard medication as a control condition. The present study aims to determine the efficacy of acupuncture in the symptomatic treatment for FM in comparison to the commonly-used medications.

## Methods

### Patients

Participants in the present study were 16 patients who were diagnosed with FM by specialists in respective hospitals and visited Acupuncture and Moxibustion Center, Meiji University of Integrative Medicine (Kyoto, Japan), seeking ways in relieving symptoms. Inclusion criteria were: (1) having met the American College of Rheumatology criteria for the diagnosis of FM for at least one year; (2) widespread pain for six months or more; (3) normal neurological examination findings of nerve function, including deep tendon reflexes, voluntary muscle action and sensory function; and (4) failure to respond to medications prescribed by FM specialists. Exclusion criteria were: (1) sufficient knowledge of acupuncture which may prevent blinding (e.g. having received acupuncture previously); (2) known bleeding diathesis; (3) having autoimmune or inflammatory diseases; (4) participation in other clinical trials; (5) pregnancy or lactation; or (6) receiving disability payments or involved in litigation related to FM. However, patients receiving FM medications were included if there had been no change in their medications for one month or longer prior to recruitment.

Patients having signed a written informed consent were recruited and randomly assigned with a computerised randomisation program (SAMPSIZE V2.0, Blackwell Sience Ltd, UK, permutated block randomization) to either group A where patients received acupuncture treatment followed by a control period or group B where they received continuous acupuncture. This study was approved by the Ethics Committee of Meiji University of Integrative Medicine.

**Figure 1:**
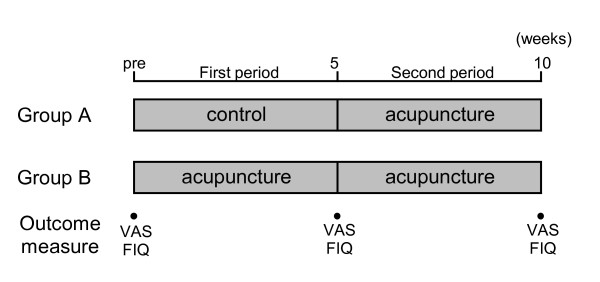
**Treatments and measurements in this trial**.

The two groups received a total of five (group A) or ten (group B) acupuncture treatments once a week. Each session lasted for 30 minutes. Group A (control) received a total of five acupuncture treatments after the control period of five weeks. Patients in group A received clinical examinations once or twice a week for over five weeks by a FM specialist prior to the intervention period whereas those in group B received a total of 10 acupuncture treatments (Figure 1).

### Blinding

Outcome measures were performed by an independent investigator who was not informed of the treatment sequence or the treatment the patient had received prior to each measurement.

### Intervention

Electroacupuncture and trigger point acupuncture were used to treat the patients. Patients received 15 minutes of electroacupuncture and then 15 minutes of trigger point acupuncture. Four pairs of electrodes were placed on the forearms and lower legs of the patients who were connected to a pulse generator (OhuPulser LFP-4000A, Zen Lryoki Corp, Japan). The current was rectangular with a biphasic top and a frequency of 4 Hz. Intensity of the current was set between the perception and pain thresholds inducing a visible muscular contraction. Disposable stainless steel needles (0.2 mm × 40 mm, Seirin, Japan) were inserted into the skin over the point to a depth of 5-20 mm. Depth of insertion was determined according to the patient's needling sensation of the specific site. Both sides of four common acupuncture points were used for patients receiving electroacupuncture.

Up to ten additional sites were chosen according to the patient's symptoms and pain pattern as well as the empirical choice of trigger point in pain treatment. Disposable stainless steel needle (0.2 mm × 40 mm, Seirin, Japan) was inserted into the skin over the trigger point to a depth of 10-20 mm, appropriate to the muscle (Table 1), attempting to elicit a local muscle twitch response using the 'sparrow pecking' technique. After the local twitch response or a reasonable attempt, the needle was retained for ten more minutes.

**Table 1:** Muscles treated in the two trigger point acupuncture groups

Muscle	Group A	Group B
**Sternocleidomastoideus**	5*	5
**Trapezius**	6	5
**Pectoralis major**	5	5
**Quadratus lumborum**	4	4
**Erector spinae**	6	5
**Gluteus medius**	5	4
**Hamstrings**	4	3
**Other**	5	4

*number of the patients of which the indicated muscle was stimulated with the acupuncture needle (summation of these numbers is not equal to the total number of the participants because of duplicated counting)

The acupuncture was performed by an acupuncturist who had four years of acupuncture training and clinical experience of three or ten years.

### Evaluation

Primary outcome measures included pain intensity quantified with a 10 cm visual analogue scale (VAS) and pain disability measured with the fibromyalgia impact questionnaire (FIQ) [24] on physical function, work, wellbeing. FIQ includes VAS for pain, sleep, fatigue, stiffness, anxiety and depression. Total score ranges between 0-100 with a higher score indicating a negative impact.

The VAS and FIQ measures were completed by the patients immediately before each treatment and analysed immediately before the first treatment (pre), five and ten weeks after the first treatment.

### Statistical analysis

Outcome measures are reported as medians and inter-quartile ranges. Mann-Whitney U test was used to analyse differences between groups at each time period. A difference was considered statistically significant when the *P *value was less than 0.05. The success of blinding was analysed by Fisher's exact test. Statistical package SYSTAT 12 (SYSTAT Software Inc, USA) was used to perform all the statistical analyses in this study.

## Results

### Patient characteristics

**Figure 2:**
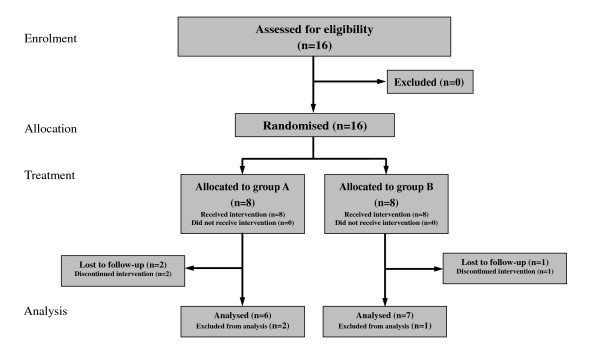
**Participants flow in the study**.

Sixteen participants (13 women and 3 men aged 25-63 years) were randomised (Figure 2). No statistically significant differences in terms of age, pain duration, VAS and treatments received were found between the two groups at baseline (Table 2).

**Table 2:** Characteristics of patients in the three groups

	Group A	Group B
**Sample size**	7	6
**Age**	45.7 (15.2)	47.3 (13.3)
**Pain duration (y)**	3.9 (3.9)	4.4 (2.3)
**VAS (mm)**	74.2 (8.4)	77.9 (10.1)
**FIQ**	64.3 (6.4)	66.3 (11.0)
**Treatments received:**		
**Amitriptylin**	4	4
**SSRIs**	2	3
**SNRIs**	2	1

Data are expressed as mean (standard deviation)

Two patients in group A and one patient in group B dropped out due to a lack of response to treatments. The dropout rates were not significantly different between the groups (*P *= 0.52, Fisher's exact test). Analyses were performed on the 13 patients who did complete the study.

### VAS scores

**Figure 3:**
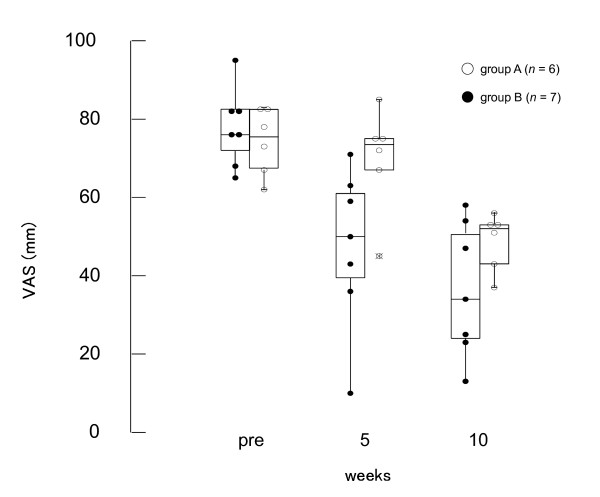
**Effects of acupuncture on VAS score**. There was a significant difference between group A and B (*U *= 25.0, *P *= 0.022), while no significant difference between groups at baseline (*U *= 5.0, *P *= 0.566) and tenth week (*U *= 13.0, *P *= 0.252).

While the median VAS score in group A remained unchanged, those in group B decreased by the fifth week of treatment (Figure 3). However, the VAS score in group A began to decrease after the fifth week when the patients in this group also started receiving acupuncture treatment. There was a significant difference in the VAS scores between groups A and B at the fifth week (*U *= 25.0, *P *= 0.022), whereas there was no significant difference between the groups at baseline (*U *= 5.0, *P *= 0.566) and at the tenth week (*U *= 13.0, *P *= 0.252) (Figure 3 and Table 3).

**Table 3:** Pain intensity VAS scores

Week	Group A	Group B
**Pre**	75.5 (68.5-81.0)	77.8 (72.0-82.5)
**5**	73.5 (68.3-75.0)	47.4 (39.5-61.0)*
**10**	52.0 (43.0-53.0)	36.3 (24.0-50.5)

Data are medians (interquartile ranges). * *P *< 0.05.

### QoL impairment

**Figure 4:**
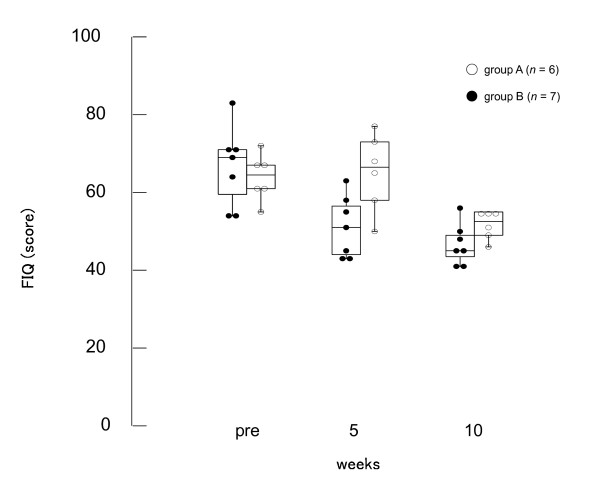
**Effects of acupuncture on FIQ score**. There was a significant difference between group A and B at the fifth week (*U *= 24.5, *P *= 0.026), while no difference between at baseline (*U *= 5.5, *P *= 0.616) and tenth week (*U *= 9.0, *P *= 0.086).

While the FIQ score decreased after acupuncture treatments in group B for five weeks, those in group B remained unchanged. However, FIQ scores in both groups A and B decreased after the fifth week when patients in both groups received acupuncture. There was a significant difference between groups at the fifth week (*U *= 24.5, *P *= 0.026), whereas there was no difference between the groups at baseline (*U *= 5.5, *P *= 0.616) and at the tenth week (*U *= 9.0, *P *= 0.086) (Figure 4 and Table 4).

**Table 4:** Fibromyalgia Impact Questionnaire scores

Week	Group A	Group B
**Pre**	64.5 (61.3-67.0)	66.7 (59.5-71.0)
**5**	66.5 (59.8-71.8)	51.1 (44.0-56.5) *
**10**	52.5 (49.5-54.8)	46.7 (43.5-49.0)

Data are medians (interquartile ranges). * *P *< 0.05.

## Discussion

The present study demonstrated a statistically significant difference between the acupuncture and active control (standard medication) groups five weeks after the first treatment. Moreover, the additionally performed acupuncture treatment after the fifth week in the control group resulted in the further reduction of the FM symptoms. These results suggest that acupuncture treatment is capable of giving additional improvement to the standard medication in the treatment of FM.

The importance of randomised controlled trials (RCT) to study the placebo effect of acupuncture was debated [25,26]. Acupuncture RCTs with various control methods such as no-treatment controls [17], non-penetration needling [18], minimal acupuncture [19,20] and mock transcutaneous electrical nerve stimulation (TENS) [21,22] have been carried out. However, positive results were often obtained in studies with a non-acupuncture control [26,27], whereas negative results tended to be from those with sham acupuncture or mock TENS [28,29].

Moreover, most studies on the efficacy of acupuncture to treat FM were uncontrolled case series with electroacupuncture treatment [1,11]. As electroacupuncture involves perceptible current, sham acupuncture as control may not work for electroacupuncture. In fact, Targino *et al*. reported that addition of acupuncture to usual treatment for FM was beneficial for pain and QoL [16]. Our study also demonstrated that utilization of standard treatment as a control may be an ideal method to evaluate efficacy of acupuncture on FM. Although there were three patients who could not complete the study protocol, we consider the number had not much impact on the results because the numbers of withdrawal were similar between groups (2 out of 8 in group A and 1 out of 8 in group B). One of the limitations of the present study is relatively small sample size. Larger scale studies are required in the future.

## Conclusion

The present study suggests that acupuncture treatment is effective to relieve pain for FM patients in terms of QoL and FIQ. Further larger scale clinical trials are warranted to confirm the findings of this study.

## Abbreviations

RCT: randomised clinical trial; FM: Fibromyalgia; QoL: Quality of Life; VAS: visual analogue scale; FIQ: Fibromyalgia Impact Questionnaire; CAM: Complementary and alternative medicine; ACR: American College of Rheumatology; TENS: transcutaneous electrical nerve stimulation

## Competing interests

The authors declare that they have no competing interests.

## Authors' contributions

KI designed the clinical study, acupuncture and wrote the manuscript. HK designed and performed the statistical data analyses. Both authors read and approved the final version of the manuscript.
